# Association between muscle quality index and periodontal disease among American adults aged ≥ 30 years: a cross-sectional study and mediation analysis

**DOI:** 10.1186/s12903-023-03520-y

**Published:** 2023-11-24

**Authors:** Jukun Song, Yadong Wu, Hong Ma, Junmei Zhang

**Affiliations:** 1 Department of Oral and Maxillofacial Surgery, the Affiliated Stomatological Hospital of Guizhou Medical University, Guizhou, 550002 China; 2 Department of Orthodontics, the Affiliated Stomatological Hospital of Guizhou Medical University, Guiyang, China

**Keywords:** MQI, Periodontitis, American adults, NHANES, Alcohol use

## Abstract

**Objective:**

The muscle quality index (MQI) is a measurement of muscle quality that is directly related to overall health. There has been little study on the relationship between the muscle quality index and periodontitis in American people beyond 30 years. Therefore, this study aimed to explore the link between periodontitis and Muscle quality index (MQI) in older Americans.

**Methods:**

Three thousand two hundred fifty-eight individuals (aged 30 to 59) who participated in the National Health and Nutrition Examination Survey (NHANES) 2011–2014 were considered eligible for the cross-sectional investigation. A hand dynamometer was used to determine the handgrip strength (HGS). Dual-energy X-ray absorptiometry was employed to calculate ASM (DXA). MQIArm was calculated by dividing the dominant hand’s HGS by the dominant arm’s ASM (in kg/kg). MQIApp was calculated by dividing the dominant hand’s HGS by the ASM (in kg/kg). MQItotal was calculated by dividing the sum of the dominant and non-dominant hands by the ASM (in kg/kg). To investigate the link between muscle quality index and periodontal disease, the weighted multivariable logistic regression models were used. Using generalized additive models, it was determined if a nonlinear connection existed. Then, we developed a two-piece linear regression model and calculated the inflection point using a recursive approach. A mediation study was performed to determine how much of the impact of MQItotal on periodontitis was mediated by potential variables.

**Results:**

Three thousand two hundred fifty-eight participants from the United States were enrolled. The OR (95% CI) for the relationship between MQItotal and periodontitis in the regression model with fully adjusted variables was 0.69 (0.53–0.91), for the connection between MQIArm and periodontitis was 0.90 (0.84–0.97), and for the association between MQIApp and periodontitis was 0.49 (0.30–0.80). MQItotal and periodontitis were shown to have a J-shaped relationship with a change point of 3.64. Before the change point, the OR (95% CI) was 0.69 (0.58, 0.82). In the analysis of drinking and married status, the interaction was statistically significant. Analysis of mediation showed that alcohol use was responsible for 0.4% (0.10 to 1.2) of the effect of MQItotal on periodontitis.

**Conclusion:**

In American adults aged over 30, the Muscle Quality Index (MQI) exhibited an independent negative correlation with moderate to severe periodontitis, demonstrating a J-shaped relationship. Furthermore, alcohol consumption may act as a mediator in the association between MQI and periodontitis.

**Supplementary Information:**

The online version contains supplementary material available at 10.1186/s12903-023-03520-y.

## Introduction

Periodontal disease, often termed gum disease, is a complex inflammatory condition instigated by bacterial plaque microorganisms. These pathogens compromise the oral environment, leading to the degradation of crucial structures such as the alveolar bone and periodontal ligaments [1, 2]. Periodontal disease seriously affects the masticatory function of the patient [3, 4], and since it is the leading reason for tooth loss, it is being monitored in many nations [5]. The National Health and Nutrition Examination Survey is the only source of nationally representative data on periodontal disease, and it was reported that 42.2–46% of American adults aged ≥ 30 years have periodontitis, of which 7.8–8.9% have severe periodontitis [6].

Both the strength and the bulk of the muscles (sarcopenia components) decrease with increasing years [7, 8]. Muscular strength per unit of muscle mass is known as the muscle quality index (MQI), and it is used to predict the risk of mortality and disability [9, 10]. Handgrip strength (HGS) is calculated by dividing the amount of skeletal muscle in the appendicular region (ASM) [11], and by measuring HGS with muscle mass in the arms (lean soft tissue) [11, 12]. Results from the little study on the connection between HGS and periodontitis are inconsistent [13–15]. The HGS was negatively linked with periodontitis in the Korean population [13, 15], but not in the American population [14]. Also, one study from South Korea looked at the link between HGS and periodontitis, and when confounding factors were taken into account, the link was not significant [13]. These studies only explored the effect of HGS on the risk of periodontitis and seldom used the comprehensive new muscle quality index [16]. Therefore, the purpose of the present research was to examine the link between muscle quality index and moderate/severe periodontitis after controlling for possible confounders among the American adult population.

## Materials and methods

### Study participants

We used cross-sectional data from the National Health and Nutrition Examination Survey for the years 2011 to 2014 (NHANES). Survey samples need to be representative of the whole US population. The survey data will be used to estimate the prevalence of different diseases and risk factors. To gather data, personal structured interviews were conducted at home, health examinations were conducted in a mobile examination center, and specimens were analyzed in the laboratory. NHANES was approved by the National Center for Health Statistics Research Ethics Review Board. Data may be accessed for free on the Centers for Disease Control and Prevention (CDC) website (https://wwwn.cdc.gov/nchs/nhanes/Default.aspx).

**Fig. 1:**
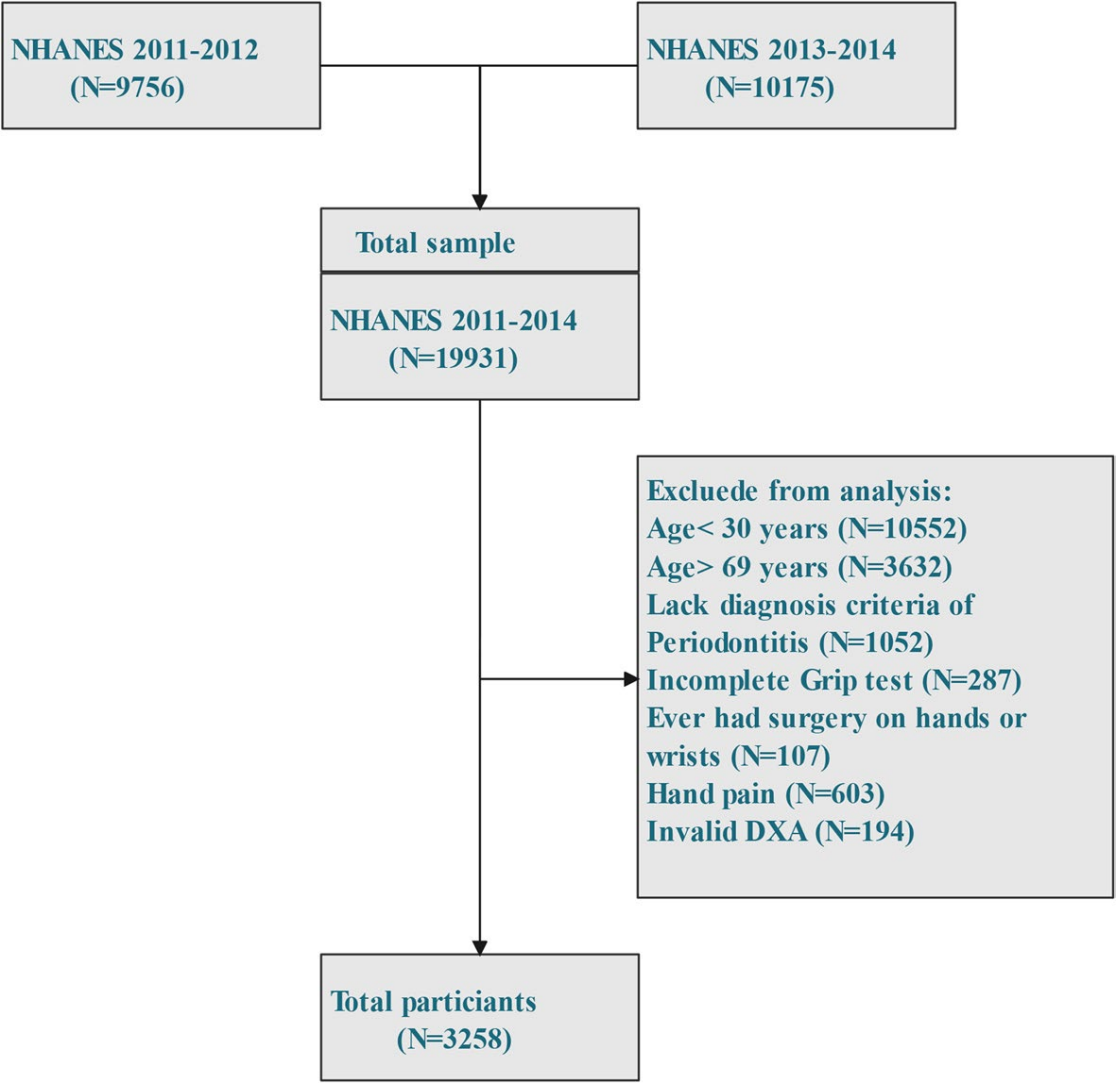
Flowchart of sample selection for the 2011–2014 NHANES

The data collection was compiled from two successive NHANES cycles, 2011–2012 and 2013–2014. Among the participants in NHANES (2011–2014) (19,931 individuals), participants were selected for the research based on the following criteria: (1) age ≥ 30 years; (2) complete handgrip test; (3) diagnosis criteria of periodontitis; and (4) the lack of rheumatoid arthritis, which is a group of disorders that may affect a person’s ability to hold on to things with their hands. Due to a lack of body composition data for individuals older than 60 years old, males and females between the ages of 30 and 59 were included in the study. The lack of HGS tests from both hands, the absence of DXA data, the presence of hand discomfort or stiffness, and surgery on either hand were our criteria for excluding participants. A flow chart is displayed in Fig. 1.

### Study variables

#### Muscle quality index

DXA, which was calibrated daily, was used to assess body composition, and findings were obtained using HologicQDR-4500 software, version Apex 3.2. (Hologic, America). Before the DXA examination, volunteers were told to refrain from using contrast or radiation in any other procedures. Body fat mass (kg and %), total lean tissue (kg), arm ASM mass (kg), and leg ASM mass were the data sources utilized in this investigation. To be consistent with the literature, we referred to lean soft tissue from limbs as adipose soft tissue (ASM). Notably, DXA-derived lean soft tissue mass is considered non-fat and non-bone tissue [7]. ASM was defined as the total of lean soft tissue from four limbs; ASM index (ASMI) was determined as the total of ASM from both arms and legs (kg)/height2 (m2) [17]. Additional information on the DXA methods may be detected on the NHANES website (https://wwwn.cdc.gov/Nchs/Nhanes/2013-2014/DXX_H.htm).

A Takei dynamometer was used to measure HGS (TKK 5401; Takei Scientific Instruments, Tokyo, Japan). The participants stood with their wrists in a neutral position and their arms extended straight down. They were told to apply maximum force on the dynamometer. Each measurement was taken three times, with a 60-s break in between, for both the dominant and non-dominant hands. The dominating hand’s greatest value was employed (https://www.cdc.gov/nchs/data/nhanes/ms.pdf). The ratio of the dominant arm’s HGS to its ASM is known as MQIArm (kg/kg)10. MQIApp (kg/kg) was computed as the ratio of dominant HGS to ASM [16], and MQItotal (kg/kg) was determined by dividing the total HGS (values from both hands) by the average weight of each hand [18].

#### Periodontitis

A dental examiner conducted the periodontal examination at the mobile Examination Center. Adults over the age of 30 years were eligible for a whole periodontal evaluation, which contained an evaluation of attachment loss (AL) and probing depth (PD). Based on the results of the NHANES survey, the CDC and the American Periodontal Association (AAP) have come up with four grades for the cases of periodontitis monitored by PD and AL: none, mild, moderate, and severe [19]. Two interproximal sites with AL measuring 6 mm (not on the same tooth) and one interproximal site with PD measuring 5 mm were considered severe periodontitis. Two interproximal sites with AL distances of 4 mm (not on the same tooth) or two interproximal sites with PD distances of 5 mm (not on the same tooth) were considered moderate periodontitis. PPD ≥ 4 mm in two or more interproximal locations (or PD ≥ 5 mm in one location) and CAL at least 3 mm but not ≥ 4 mm in two or more interproximal locations were considered mild periodontitis [19]. The participants were dichotomized as no/mild periodontitis and moderate/severe periodontitis [14, 20].

#### Other covariables

The self-reported participants’ demographic information, including age, gender, marital status, household income, and education level via the initial screening questionnaire. The population was divided into the following categories: White non-Hispanics, Mexican Americans, Asian non-Hispanics, Black non-Hispanics, and others (Including Multi-Racial and Other Hispanic populations). Family poverty-income ratio (PIR) data were used to categorize household income according to its relationship to poverty and divided into three categories: low income (PIR 1.3), medium income (1.3 < PIR < 3.), and high income (> 3.5) [21]. The education level was categorized as less than ninth grade, ninth to eleventh grade, high school graduate, some college or associate (AA) degree, and college graduate or higher. The body mass index (BMI) was imputed using the following formula: body mass (kg)/height squared (m2) [22]. Smokers were divided into three categories: never-smokers, current smokers (those who have smoked more than 100 cigarettes in their lifetime), and past smokers (those who have smoked more than 100 cigarettes but have given up). Alcohol use was categorized as never, former, mild, moderate, and heavy. The diagnosis of chronic kidney disease and cardiovascular disease, rheumatoid arthritis, and diabetes was determined by the physician’s report of whether the individual had chronic kidney disease, cardiovascular disease, rheumatoid arthritis, or diabetes.

### Statistical analysis

Due to the intricate nature of the survey design, the statistical analysis takes into account the sample weight for analysis following CDC recommendations. For accurate national population estimates, sample weights and main sampling units were used in all analyses. The participants were classified into four groups for baseline information using the MQI quartiles. Continuous variables are expressed as survey-weighted mean (95% CI) and categorical variables as percentages (95% CI). The baseline characteristics for continuous and categorical variables were compared using weighted linear regression and weighted Chi-square analysis, respectively. Using weighted multivariable logistic regression equations, we evaluate the connection between MQI and periodontitis. Linear trend tests were used to assess the consistency of the association. The non-linear association was then investigated using generalized additive models (GAMs) and smooth curve fittings. The inflection point was inferred from the smoothing curve using a recursive method, which was used by two-piecewise linear regression models. Adjusting the model for the aforementioned possible confounders, we conducted subgroup analyses and interaction analyses for categorical covariables such as age, gender, CVD, CKD, DM, Hypertension, alcohol consumption, smoking, and BMI. Last but not least, we did a mediation analysis using the product of coefficients method and compared the indirect effect of MQItotal on periodontitis through alcohol consumption to the total effect of MQItotal on periodontitis [23, 24]. All analyses were conducted using R software. A P value less than 0.05 was considered statistically significant.

## Result

### Baseline characteristics

A total of 3,258 individuals, with a mean age of 44.51 ± 9.6 years, were considered eligible for the study. In all, the weighted average MQIArm (kg/kg), MQIApp (kg/kg), and MQItotal (kg/kg) values were 12.58, 1.73, and 3.37 kg/kg, respectively. And 37.45% of participants had moderate/severe periodontitis. The most of individuals were males (50.58%) and white (41.34%) non-Hispanics, with a mean BMI (kg/m2) of 29.24 6.53 kg/m2. The majority of the participants claimed to have more than 12 years of education (72.16%) and a high income (38.0%). Based on the MQI.total quartiles, Table 1 displays the weighted distribution of population features and variables. The MQI quartiles revealed no significant differences in periodontitis, cardiovascular disease, smoking, alcohol intake, or marital status. The participants in the group with the greatest MQItotal (Q4) have a higher probability of becoming male, younger, non-Hispanic white, have a greater level of education, a higher income, a lower BMI, and a decreased chance of developing diabetes.

**Table 1:** Weighted characteristics of study participants based on MQItotal quartiles (*N* = 3258)

Variables	Total sample	Q1	Q2	Q3	Q4	*P* value
Age	44.51 (44.08,44.94)	45.88 (45.13,46.63)	45.07 (44.21,45.93)	43.64 (42.68,44.59)	43.55 (42.57,44.52)	0.003
MQI.total	3.37 (3.33,3.41)	2.58 (2.56,2.60)	3.16 (3.15,3.17)	3.58 (3.56,3.59)	4.13 (4.11,4.15)	< 0.0001
MQI.arm	12.58 (12.43,12.73)	10.17 (10.03,10.32)	11.91 (11.75,12.08)	13.28 (13.13,13.44)	14.82 (14.65,14.99)	< 0.0001
MQI.app	1.73 (1.71,1.75)	1.33 (1.31,1.34)	1.62 (1.61,1.63)	1.84 (1.83,1.85)	2.12 (2.11,2.13)	< 0.0001
poverty	3.18 (3.02,3.34)	2.99 (2.85,3.13)	3.20 (2.99,3.42)	3.28 (3.07,3.49)	3.23 (3.00,3.47)	0.16
BMI_kg.m2	29.24 (28.84,29.64)	34.41 (33.76,35.06)	30.09 (29.52,30.66)	27.48 (27.08,27.87)	25.28 (24.98,25.59)	< 0.0001
coffee.gram	344.45 (318.29,370.61)	292.23 (249.11,335.35)	355.50 (323.17,387.82)	370.12 (314.81,425.43)	355.44 (293.79,417.08)	0.17
Sex, N(%)						< 0.0001
Female	1679 (49.41)	517 (28.58)	416 (25.26)	379 (24.54)	367 (21.62)	
Male	1719 (50.59)	334 (18.73)	431 (26.35)	471 (27.85)	483 (27.08)	
Race, N (%)						< 0.0001
Mexican American	420 (12.36)	112 (24.83)	101 (24.20)	99 (24.38)	108 (26.59)	
Non-Hispanic Asian	491 (14.45)	53 (10.46)	100 (20.80)	141 (28.45)	197 (40.29)	
Non-Hispanic Black	715 (21.04)	275 (38.29)	175 (24.59)	151 (21.00)	114 (16.12)	
Non-Hispanic White	1379 (40.58)	311 (22.03)	366 (26.66)	360 (27.03)	342 (24.27)	
Other	393 (11.57)	100 (23.55)	105 (25.74)	99 (27.20)	89 (23.51)	
Marital status, N(%)						0.35
Living with partner	276 (8.12)	70 (22.17)	69 (28.09)	66 (22.39)	71 (27.35)	
Married	2633 (77.49)	629 (22.78)	661 (26.04)	664 (26.57)	679 (24.61)	
Never married	489 (14.39)	152 (29.09)	117 (23.11)	120 (26.18)	100 (21.62)	
MQI.armQ, N(%)						< 0.0001
Q1	850 (25.01)	575 (65.08)	241 (31.54)	26( 2.50)	8( 0.89)	
Q2	847 (24.93)	197 (21.70)	326 (38.44)	274 (33.91)	50( 5.95)	
Q3	852 (25.07)	70( 8.49)	194 (22.79)	328 (39.37)	260 (29.34)	
Q4	849 (24.99)	9( 1.45)	86 (10.85)	222 (27.27)	532 (60.43)	
MQI.appQ, N(%)						< 0.0001
Q1	849 (24.99)	735 (85.71)	112 (14.14)	2( 0.15)	0( 0.00)	
Q2	852 (25.07)	110 (10.99)	621 (74.98)	121 (14.03)	0( 0.00)	
Q3	847 (24.93)	5( 0.67)	111 (13.17)	626 (73.26)	105 (12.90)	
Q4	850 (25.01)	1( 0.04)	3( 0.17)	101 (12.73)	745 (87.05)	
Periodontitis, N(%)						0.14
No	2030 (59.74)	474 (22.67)	487 (24.43)	554 (28.38)	515 (24.52)	
Mild	90 (2.65)	25 (23.71)	26 (29.27)	22 (26.12)	17 (20.90)	
Moderate	977 (28.75)	270 (25.62)	259 (27.85)	208 (21.99)	240 (24.54)	
Severe	301 (8.86)	82 (24.23)	75 (30.73)	66 (20.70)	78 (24.34)	
CVD, N(%)						0.13
No	3278 (96.47)	799 (23.14)	821 (25.92)	823 (26.34)	835 (24.60)	
Yes	120 (3.53)	52 (36.30)	26 (22.40)	27 (22.73)	15 (18.57)	
CKD, N(%)						0.02
No	2949 (90.24)	702 (22.52)	733 (25.93)	744 (26.65)	770 (24.90)	
Yes	319 (9.76)	120 (33.44)	80 (25.67)	68 (19.73)	51 (21.15)	
Menstrual status, N(%)						0.49
No	175 (30.49)	62 (34.02)	38 (21.77)	45 (26.31)	30 (17.89)	
Yes	399 (69.51)	149 (34.21)	114 (27.83)	72 (19.66)	64 (18.30)	
DM, N(%)						< 0.0001
DM	433 (12.74)	186 (42.14)	121 (31.11)	86 (19.11)	40( 7.64)	
IFG	115 (3.38)	31 (28.58)	36 (32.67)	24 (25.60)	24 (13.15)	
IGT	128 (3.77)	29 (24.46)	29 (27.24)	30 (24.27)	40 (24.03)	
No	2722 (80.11)	605 (20.94)	661 (24.80)	710 (27.24)	746 (27.02)	
Hypertension, N(%)						< 0.0001
No	2276 (66.98)	460 (19.71)	541 (24.52)	615 (28.61)	660 (27.16)	
Yes	1122 (33.02)	391 (31.66)	306 (28.56)	235 (21.18)	190 (18.60)	
Smoke, N(%)						0.1
Former	678 (19.96)	165 (21.37)	183 (29.80)	177 (27.95)	153 (20.89)	
Never	1961 (57.73)	509 (24.47)	493 (25.81)	485 (25.38)	474 (24.34)	
Now	758 (22.31)	177 (23.32)	171 (21.50)	187 (26.65)	223 (28.52)	
Alcohol user, N(%)						0.48
Never	394 (12.17)	121 (31.10)	98 (23.81)	79 (21.88)	96 (23.21)	
Former	412 (12.73)	110 (21.85)	94 (26.27)	108 (25.83)	100 (26.05)	
Mild	1129 (34.88)	260 (22.36)	291 (26.70)	307 (27.61)	271 (23.34)	
Moderate	586 (18.1)	141 (22.72)	158 (26.75)	135 (25.00)	152 (25.52)	
Heavy	716 (22.12)	184 (24.44)	165 (24.14)	185 (27.59)	182 (23.83)	
Educational level, N(%)						0.04
Less than 9th grade	182 (5.36)	42 (20.01)	47 (27.73)	46 (27.03)	47 (25.23)	
9-11th grade	388 (11.42)	106 (26.85)	85 (21.35)	86 (23.48)	111 (28.32)	
High School graduate	710 (20.89)	192 (26.92)	169 (23.26)	168 (23.19)	181 (26.63)	
Some college or AA degree	1032 (30.37)	294 (25.76)	263 (27.04)	252 (26.40)	223 (20.80)	
College graduate or above	1086 (31.96)	217 (19.29)	283 (27.10)	298 (28.37)	288 (25.23)	

### Association between MQI and periodontitis

The link between MQI and the risk of periodontitis is shown in Table 2. As the adjusted variables were altered, the correlation weakened but was maintained. After adjusting for age, sex, race, marital status, and education level, the risk of periodontitis was significantly reduced. After adjusting for all confounding factors, the OR remain significant. When MQI was analyzed as a continuous variable in the whole adjusted logistic regression analysis model, a statistically significant link between MQIApp, MQIArm, and MQItotal and the risk of periodontitis was observed (MQItotal: OR 0.69, 95% CI 0.53–0.91, *P* = 0.0078; MQIApp: OR 0.49, 95% CI 0.30–0.80, *P* = 0.0049; MQIArm: OR 0.90, When the MQI was analyzed as categorical variables, the OR for the Q2, Q3, and Q4 groups compared to the Q1 group was still significant.

**Table 2:** Association between MQItotal with periodontitis in different models

Exposure	Non-adjusted	Adjust I	Adjust II
MQItotal	0.84 (0.75, 0.94) 0.0025	0.85 (0.74, 0.97) 0.0176	0.69 (0.53, 0.91) 0.0078
MQIapp	0.69 (0.56, 0.86) 0.0008	0.75 (0.58, 0.96) 0.0244	0.49 (0.30, 0.80) 0.0049
MQIarm	0.88 (0.85, 0.91) < 0.0001	0.96 (0.92, 0.99) 0.0249	0.90 (0.84, 0.97) 0.0036
MQItotal quartile			
Q1	1.0	1.0	1.0
Q2	0.92 (0.75, 1.12) 0.4022	1.01 (0.80, 1.27) 0.9322	0.75 (0.49, 1.17) 0.2075
Q3	0.67 (0.55, 0.82) 0.0001	0.74 (0.59, 0.94) 0.0146	0.68 (0.43, 1.06) 0.0884
Q4	0.82 (0.67, 1.00) 0.0539	0.86 (0.67, 1.09) 0.2070	0.57 (0.36, 0.91) 0.0177
MQIarm quartile			
Q1	1.0	1.0	1.0
Q2	0.73 (0.60, 0.89) 0.0018	0.83 (0.66, 1.04) 0.1097	0.66 (0.43, 1.00) 0.0480
Q3	0.59 (0.48, 0.72) < 0.0001	0.77 (0.61, 0.97) 0.0280	0.56 (0.36, 0.87) 0.0098
Q4	0.44 (0.36, 0.54) < 0.0001	0.75 (0.58, 0.96) 0.0208	0.48 (0.30, 0.78) 0.0034
MQIapp quartile			
Q1	1.0	1.0	1.0
Q2	0.91 (0.75, 1.11) 0.3748	1.07 (0.85, 1.35) 0.5625	0.91 (0.59, 1.41) 0.6763
Q3	0.72 (0.59, 0.88) 0.0012	0.79 (0.62, 1.00) 0.0485	0.62 (0.40, 0.97) 0.0357
Q4	0.74 (0.61, 0.91) 0.0037	0.83 (0.65, 1.06) 0.1279	0.54 (0.34, 0.85) 0.0076

Non-adjusted model adjust for: None

Adjust I model adjust for: age, sex, race, marital status, and education level

Adjust II model adjust for: age, sex, race, income to poverty ratio, education level, smoking status, alcohol consumption, marital status, BMI, CVD, menstrual status, hypertension, diabetes, and CKD

**Fig. 2:**
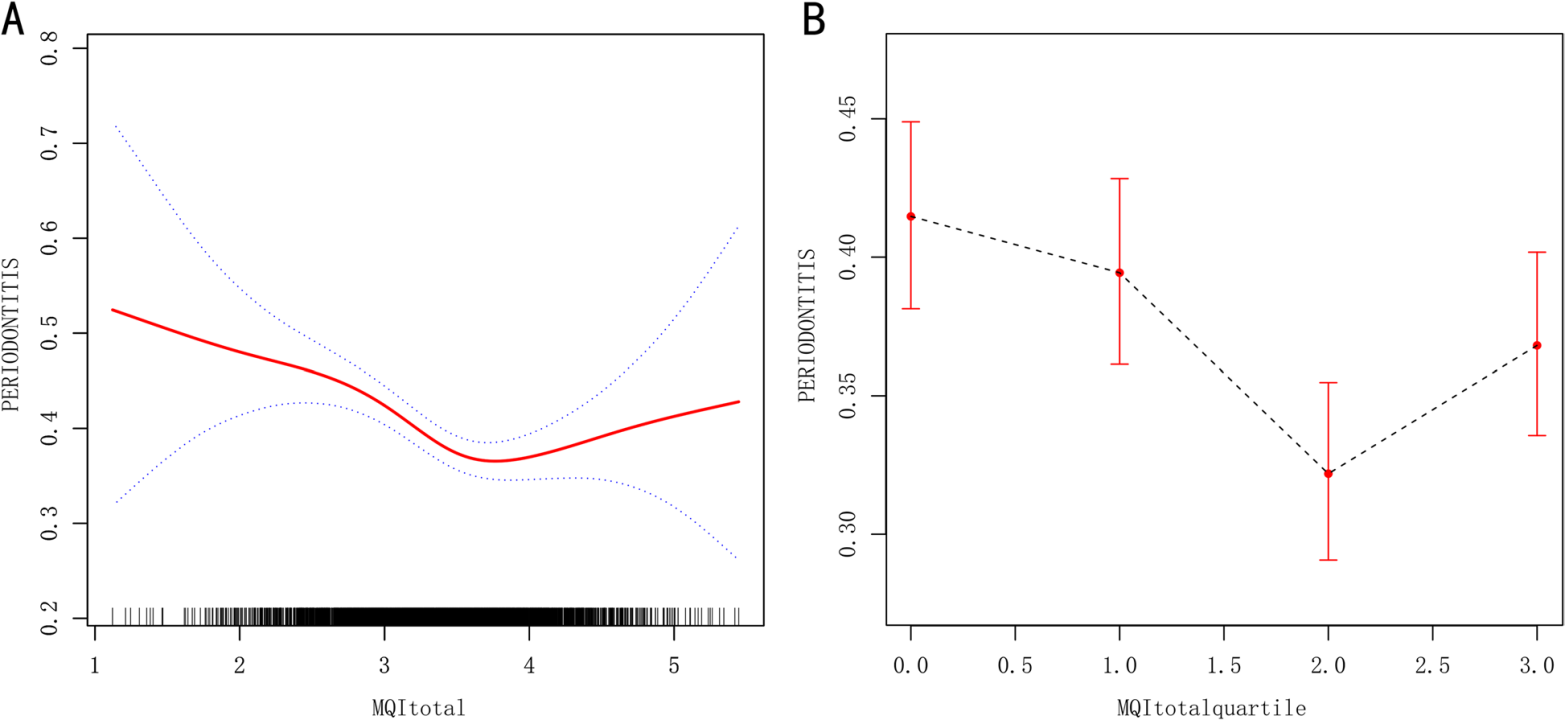
Dose–response relationship between MQItotal and periodontitis. **A** is for continuous variable of MQItotal, and **B** is categorical variable of MQItotal

**Fig. 3:**
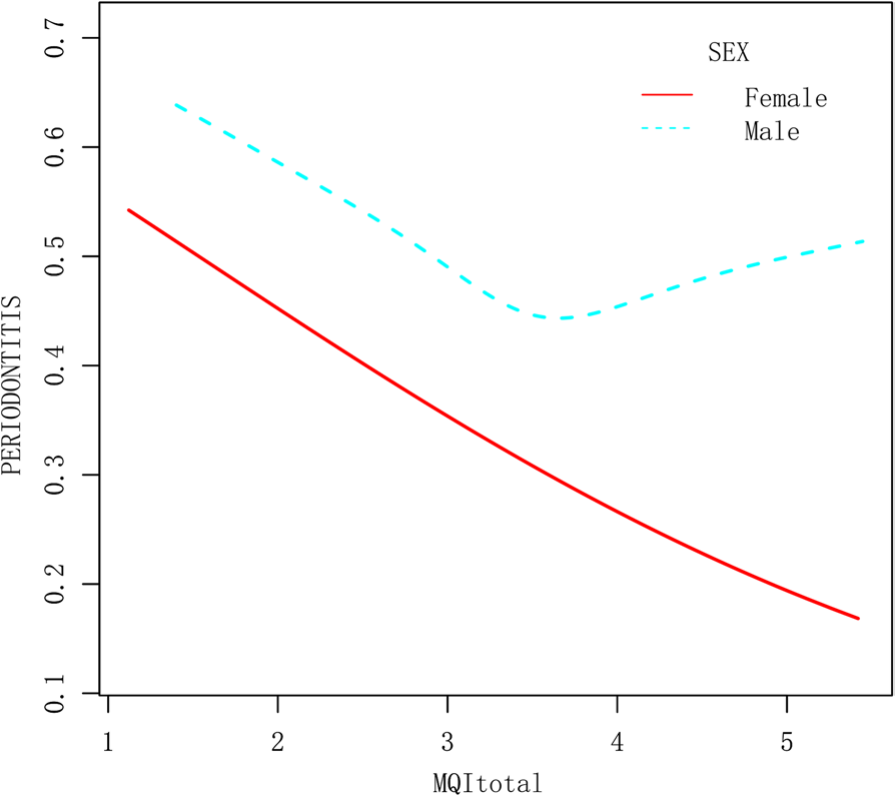
The association between MQItotal and periodontitis stratified by sex

A significant relationship between dose and response was also found (MQIApp: P for trend = 0.013, MQItotal: P for trend = 0.003), but not for MQIarm (Table 3, Supplementary Tables 1 and 2). A linear association was detected between MQIarm and periodontitis (Supplementary Fig. 1A, B). A non-linear and J-shaped link was observed by GAM and smoothing curve, with a change point of 3.64 for MQItotal (Fig. 2A, B) and 2.06 for MQIArm (Supplementary Fig. 2A, B) threshold effect analysis. Before the turning point, there was a strong link between MQItotal and periodontitis (OR: 0.69, 95%:0.58–0.82, *p* < 0.0001). After the cut-off point, however, the link was non-significant (OR 1.27, 95% CI 0.95–1.72, *P* = 0.1109). Even when the relationship was stratified by gender, the relationship remained non-linear for MQItotal (Fig. 3). Similar findings were observed in Supplementary Figs. 3 and 4.

**Table 3:** Threshold effect analysis between MQItotal and periodontitis

Outcome	Periodontitis (OR, 95%CI, P)
Fitting by weighted linear regression model	0.84 (0.75, 0.94) 0.0025
Fitting by weighted two-piecewise linear regression mode	
Inflection point	3.64
< 3.64	0.69 (0.58, 0.82) < 0.0001
≥ 3.64	1.27 (0.95, 1.72) 0.1109
Log likelihood ratio test	0.003

**Fig. 4:**
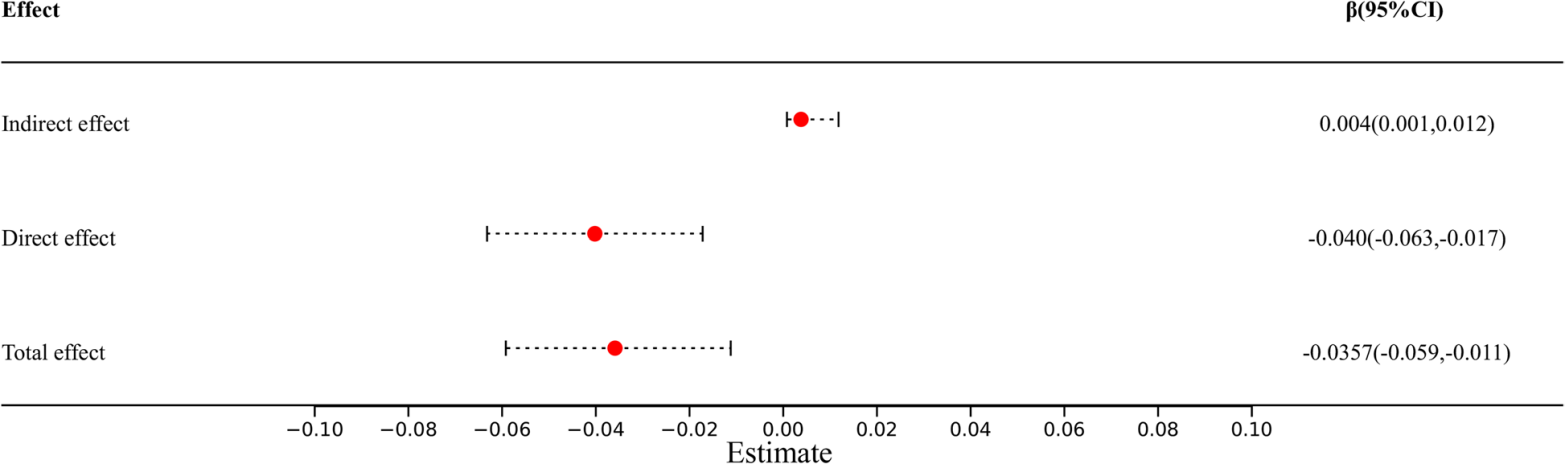
The mediation analysis for alcohol use in MQItotal and periodontitis

We also used a stratified analysis to explore whether there was a link between covariates and MQItotal and periodontitis (Table 4). There was a highly consistent negative correlation between the MQItotal and periodontitis across all subgroups. The impact of MQItotal on periodontitis was lower in people with females (OR 0.728, 95% CI 0.580–0.913, *P* = 0.008; P-interaction = 0.310). Compared with those who married or lived with a partner, those who never married had a lower risk of periodontitis (OR 0.606, 95% CI 0.444–0.828, *P* = 0.003, P-interaction = 0.008). Compared with subjects who currently smoke, those who never smoke or former smoke had a lower risk of periodontitis (never smoke: OR 0.727, 95% CI 0.583–0.906, *P* = 0.006, P-interaction = 0.100. Compared with subjects whose education level is less than 9th grade or 9-11th grade, those who obtain college graduate or above had a lower risk of periodontitis (Some college or AA degree: OR 0.665, 95% CI 0.503–0.877, *P* = 0.005; P-interaction = 0.055). Compared with subjects who smoke, those who never smoked had a lower risk of periodontitis (OR 0.616, 95% CI 0.457–0.829, *P* = 0.002, P-interaction = 0.042). Other covariates had no significant interaction on the correlation between MQItotal and periodontitis. Mediation analyses showed that 0.4% (95% CI 0.10 to 1.2; *p* = 0.01) of the observational association of MQItotal with the risk of periodontitis was mediated through alcohol use (Fig. 4).

**Table 4:** Subgroup analysis of the relationship between MQItotal and periodontitis

Variables	95% CI	*P*	P for interaction
Sex			0.31
Female	0.728 (0.580,0.913)	0.008	
Male	0.831 (0.697,0.991)	0.040	
Race			0.269
Mexican American	0.742 (0.514,1.072)	0.107	
Non-Hispanic Asian	1.089 (0.861,1.377)	0.462	
Non-Hispanic Black	1.086 (0.851,1.386)	0.493	
Non-Hispanic White	0.871 (0.678,1.119)	0.270	
Other	0.741 (0.528,1.042)	0.082	
Marital status			0.008
Living with partner	1.417 (0.983,2.043)	0.061	
Married	0.864 (0.713,1.046)	0.129	
Never married	0.606 (0.444,0.828)	0.003	
CVD			0.190
No	0.857 (0.727,1.011)	0.066	
Yes	1.318 (0.698,2.488)	0.376	
CKD			0.294
No	0.902 (0.782,1.040)	0.149	
Yes	0.731 (0.481,1.111)	0.137	
DM			0.225
DM	1.182 (0.717,1.950)	0.500	
IFG	1.352 (0.652,2.802)	0.402	
IGT	0.532 (0.233,1.216)	0.128	
No	0.886 (0.742,1.057)	0.172	
Hypertension			0.372
No	0.845 (0.701,1.018)	0.075	
Yes	0.980 (0.734,1.308)	0.885	
Smoke			0.100
Never	0.727 (0.583,0.906)	0.006	
Former	0.696 (0.460,1.054)	0.085	
Now	1.050 (0.795,1.386)	0.723	
Alcohol user			0.042
Never	0.905 (0.604,1.357)	0.618	
Former	1.068 (0.753,1.515)	0.704	
Mild	0.616 (0.457,0.829)	0.002	
Moderate	1.006 (0.730,1.386)	0.970	
Heavy	0.845 (0.658,1.086)	0.182	
Education level			0.055
Less than 9th grade	0.912 (0.484,1.717)	0.765	
9-11th grade	1.152 (0.773,1.718)	0.474	
High School graduate	1.039 (0.792,1.362)	0.778	
Some college or AA degree	0.665 (0.503,0.877)	0.005	
College graduate or above	0.756 (0.589,0.970)	0.029	
Age			0.449
30–39	0.853 (0.641,1.135)	0.266	
40–49	0.839 (0.644,1.093)	0.185	
50–59	1.026 (0.805,1.307)	0.832	
Family poverty-income ratio (PIR)			0.166
High income	0.733 (0.516,1.040)	0.080	
Low income	1.068 (0.875,1.302)	0.507	
Middle income	0.864 (0.677,1.103)	0.231	
BMI (kg.m2)			0.091
< 25	1.406 (0.942,2.100)	0.093	
> = 30	0.797 (0.581,1.092)	0.152	
25–30	0.926 (0.668,1.283)	0.634

## Discussion

The primary findings of the study demonstrated that after controlling for confounding variables, it was found that the MQI and the risk of periodontitis were independently and negatively correlated, and the relationship was nonlinear among the US population aged over 30 years old. Following curve smoothing, we found that the positive correlation in the overall US population was J-shaped and showed a dose–response effect, with 3.60 being the change point and a considerably decreased risk of periodontitis with rising MQI.total before the turning point. Higher MQI was substantially correlated with variables including age, income, marital status, BMI, and chronic medical problems.

Currently, the KNHANES research investigated the association between RHGS and periodontitis [13]. However, after controlling for all confounders, the link was determined to be nonexistent. Another NHANES research examined the link between moderate/severe periodontitis and handgrip strength in the United States, and the results demonstrated that those with moderate/severe periodontitis had considerably lower grip strength. However, after controlling for possible confounding factors, the significance of the link vanished [14]. In the current investigation, MQI (MQIApp, MQIArm, and MQItotal) was linked with a reduced risk of periodontitis in people older than 30 years. Differences in the demographic studied (South Koreans), age of respondents (below 60), MQI definition, and periodontitis case classification (community periodontal index (CPI)) may account for the conflicting findings between this research and others on the association between MQI and periodontitis. In the research, periodontitis cases are classified based on whole mouth periodontal exploration of six regions of each tooth, except the third molar, which greatly increased the diagnostic accuracy of periodontitis [19]. Meanwhile, a more objective index (MQI) was employed to reflect muscle strength [16, 18].

The following factors may help explain the study’s findings. Periodontitis is caused mostly by the deposition of dental biofilm on the tooth surface [4, 25]. Biofilm is a sticky microbial population made up of over 700 distinct bacterial species that are bound to salivary glycoproteins [26]. Toothbrushing is the most basic and effective approach for removing dental biofilm [27]. However, the efficiency of manual teeth brushing is often dependent on several parameters, including brushing motions and hand motor performance [28, 29]. Particularly, poor finger or hand joint function is said to impact the degree of dental biofilm production [30, 31]. Growing data suggest a link between dental health and physical decline and weakness in old life [32–34]. Low hand grip strength is an essential indicator of physical decline in old age and one of the frailty phenotype’s defining characteristics [35].

In addition, alcohol use may mediate the relationship between muscle strength and periodontitis. It was well-known that alcohol use acted as a risk factor for periodontitis, and there was a link between drinking alcohol and a higher risk of developing periodontitis [36–38]. At the same time, Alcohol abuse increases the risk of sarcopenia through direct and indirect mechanisms associated with poor skeletal muscle protein metabolism, according to available research [39, 40]. Consumption of alcohol may result in dysbiosis and autophagy of the gut microbiota-induced hyperammonemia, which initiates the up-regulation of muscle protein breakdown and the down-regulation of muscle protein synthesis by activating myostatin, AMPK, and REDD1, and deactivating IGF-1. These changes take place as a result of the activation of myostatin, AMPK, and REDD1 as well as the activation of IGF-1 [41, 42].

Our study presents several limitations that warrant consideration. First and foremost, due to its cross-sectional design, it is imprudent to deduce a causal relationship between MQI and moderate to severe periodontitis. Second, the NHANES data from 2011–2014 did not encompass several recognized and potential confounders. Factors such as specific inflammatory markers and variations in daily tooth brushing habits, both in frequency and duration, were not incorporated into our analysis. Lastly, our reliance on the 2012 AAP/CDC periodontitis case definition may be perceived as a limitation. Historically, this definition was a cornerstone in global epidemiological studies concerning periodontal disease [6, 19]. However, in 2018, the EFP/AAP proposed a more nuanced classification system that nuances periodontitis based on its severity, complexity, and progression rate [43, 44]. Under this novel schema, maximum loss measurements at sites of 1–2 mm are denoted as incipient (stage 1), 3–4 mm as moderate (stage 2), and ≥ 5 mm as severe (stages 3 and 4) [43]. This transition to a newer classification system may affect the comparability and interpretation of our results in the broader context of recent periodontal research.

Nonetheless, this study holds significant importance. To our knowledge, it is the first to utilize NHANES data to examine the association between MQI and moderate to severe periodontitis among American adults. Our data suggest a consistent and independent inverse relationship between MQI levels and the risk of moderate to severe periodontitis. A comprehensive understanding of this association necessitates further prospective studies, taking into account both recognized and potential confounding factors.

Based on data from two NHANES surveys (2011–2012 and 2013–2014), we observed that the Muscle Quality Index (MQI) was independently and negatively associated with periodontitis in American adults over 30 years of age. Additionally, alcohol consumption appeared to mediate the relationship between MQI and periodontitis.

### Supplementary Information

**Additional file 1: Supplementary Figure 1.** Dose-response relationship between MQIarm and periodontitis. A. is for the continuous variable of MQIarm, and B is the categorical variable of MQIarm. **Supplementary Figure 2.** Dose-response relationship between MQIapp and periodontitis. A. is for the continuous variable of MQIapp, and B is the categorical variable of MQIapp. **Supplementary Figure 3.** The association between MQIarm and periodontitis stratified by sex. **Supplementary Figure 4.** The association between MQIapp and periodontitis stratified by sex. **Supplementary Table 1. **Threshold Effect Analysis between MQIapp and periodontitis. **Supplementary Table 2. **Threshold Effect Analysis between MQIarm and periodontitis.  

## Data Availability

The data supporting the findings of this study are available from the corresponding author upon request.
