# Supplementary material for: Association between muscle quality index and periodontal disease among American adults aged ≥ 30 years: a cross-sectional study and mediation analysis
Source: BMC Oral Health. 2023 Nov 24;23:918. doi: 10.1186/s12903-023-03520-y (PMC10675865; doi:10.1186/s12903-023-03520-y)
Supplement: Supplementary file 1 — Additional file 1: Supplementary Figure 1. Dose-response relationship between MQIarm and periodontitis. A. is for the continuous variable of MQIarm, and B is the categorical variable of MQIarm. Supplementary Figure 2. Dose-response relationship between MQIapp and periodontitis. A. is for the continuous variable of MQIapp, and B is the categorical variable of MQIapp. Supplementary Figure 3. The association between MQIarm and periodontitis stratified by sex. Supplementary Figure 4. The association between MQIapp and periodontitis stratified by sex. Supplementary Table 1. Threshold Effect Analysis between MQIapp and periodontitis. Supplementary Table 2. Threshold Effect Analysis between MQIarm and periodontitis. [file 12903_2023_3520_MOESM1_ESM.docx]

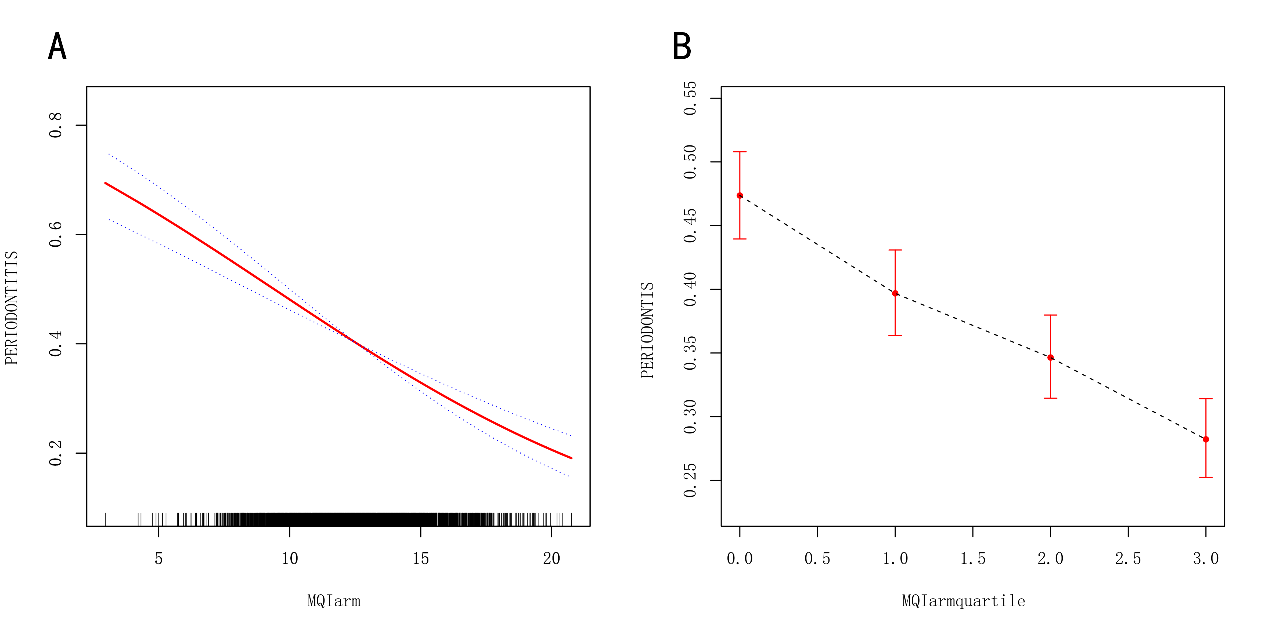


Supplementary Figure 1: Dose-response relationship between MQIarm and periodontitis. A. is for the continuous variable of MQIarm, and B is the categorical variable of MQIarm.


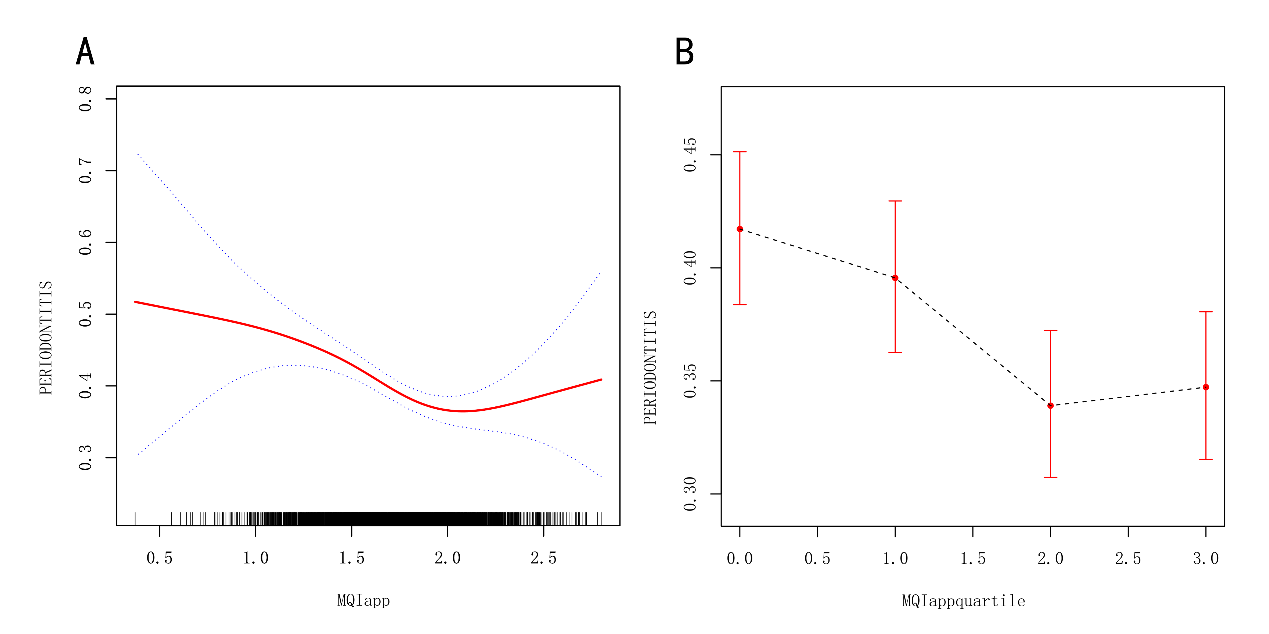


Supplementary Figure 2: Dose-response relationship between MQIapp and periodontitis. A. is for the continuous variable of MQIapp, and B is the categorical variable of MQIapp.


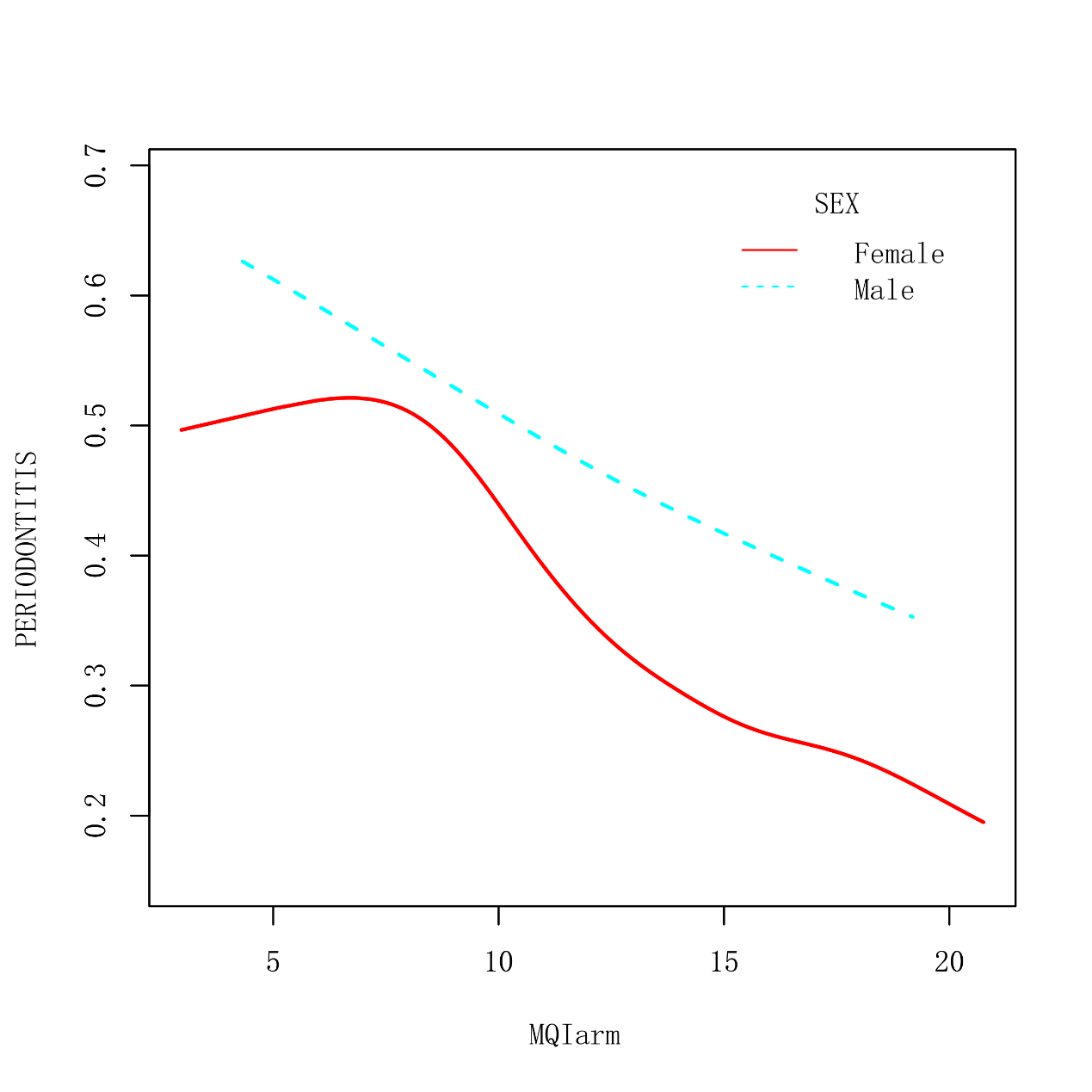


Supplementary Figure 3: The association between MQIarm and periodontitis stratified by sex.


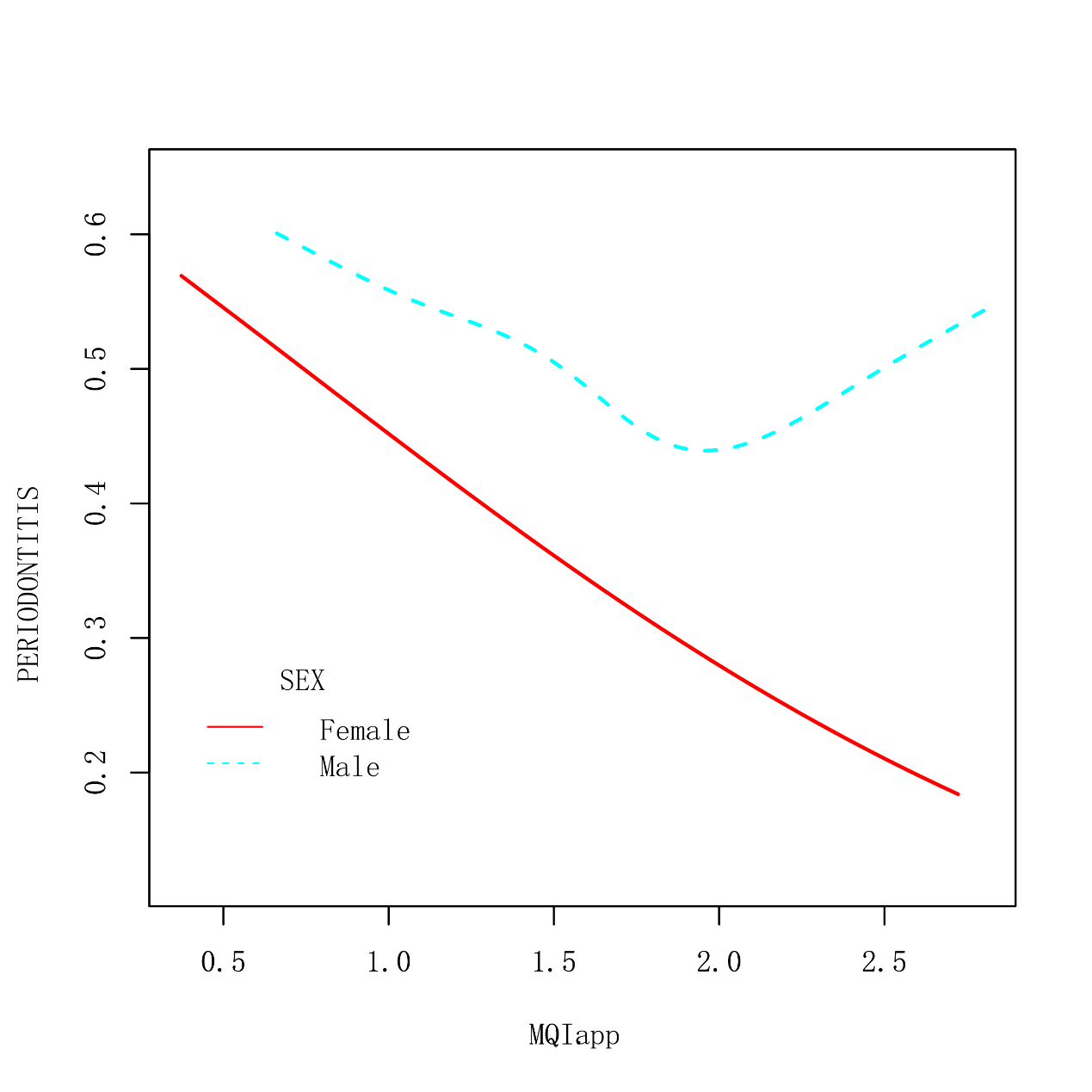


Supplementary Figure 4: The association between MQIapp and periodontitis stratified by sex.

**Supplementary Table 1. Threshold Effect Analysis between MQIapp and periodontitis.**

| **Outcome** | **Periodontitis (OR, 95%CI, P)** |
| --- | --- |
| Fitting by weighted linear regression model | 0.69 (0.56, 0.86) 0.0008 |
| Fitting by weighted two-piecewise linear regression mode |  |
| Inflection point | 2.06 |
| < 2.06 | 0.57 (0.43, 0.74) <0.0001 |
| ≥ 2.06 | 2.11 (0.86, 5.16) 0.1030 |
| Log likelihood ratio test | 0.053 |

**Supplementary Table 2. Threshold Effect Analysis between MQIarm and periodontitis.**

| **Outcome** | **Periodontitis (OR, 95%CI, P)** |
| --- | --- |
| Fitting by weighted linear regression model | 0.88 (0.85, 0.91) <0.0001 |
| Fitting by weighted two-piecewise linear regression mode |  |
| Inflection point | 15.36 |
| < 15.36 | 0.87 (0.84, 0.91) <0.0001 |
| ≥ 15.36 | 0.94 (0.81, 1.09) 0.3865 |
| Log likelihood ratio test | 0.005 |
